# Survey of Antithrombotic Treatment in Rural Patients (>60 years) with Atrial Fibrillation in East China

**DOI:** 10.1038/s41598-018-24878-y

**Published:** 2018-05-01

**Authors:** Yong Wei, Juan Xu, Haiqing Wu, Genqing Zhou, Songwen Chen, Caihong Wang, Yahong Shen, Shunhong Yang, Bin Wang, Zheng He, Jianping Sun, Weidong Sun, Ping Ouyang, Shaowen Liu

**Affiliations:** 1 Department of Cardiology, Shanghai Songjiang Central Hospital, Shanghai, 201600 China; 2 Department of Cardiology, Shanghai First People’s Hospital, School of Medicine, Shanghai Jiao Tong University, Shanghai, 200800 China; 3 Xinbang Community Health Service Center, Songjiang District, Shanghai, 201605 China; 4 Maogang Community Health Service Center, Songjiang District, Shanghai, 201605 China; 5 Chedun Community Health Service Center, Songjiang District, Shanghai, 201611 China; 6 Dongjing Community Health Service Center, Songjiang District, Shanghai, 201613 China; 7 Xiaokunshan Community Health Service Center, Songjiang District, Shanghai, 201616 China; 8 Shihudang Community Health Service Center, Songjiang District, Shanghai, 201604 China; 9 Yexie Community Health Service Center, Songjiang District, Shanghai, 201609 China

## Abstract

The prevalence and antithrombotic treatment of atrial fibrillation (AF) in Chinese rural population is not well known. The aim of this study was to investigate the extent to which antithrombotic treatment was prescribed for rural AF patients >60 years. We identified 828 AF patients from 36734 rural residents >60 years in Shanghai China. Our data indicated the overall prevalence rate of AF was 2.3% in rural population >60 years in East China and 38.9% of AF patients underwent antithrombotic therapy, including warfarin (5.9%), aspirin (29.6%), clopidogrel (2.9%) and aspirin combined with clopidogrel (0.5%). Of enrolled subjects, 98.4% had CHA_2_DS_2_-VASc score ≥1, 72.0% had HAS-BLED score <3 and 59.2% had CHA_2_DS_2_-VASc score ≥2 with HAS-BLED score <3. Missing early detection (34.9%), delay in seeking treatment for asymptomatic AF (25.5%) and doctors’s incomplete inform of AF-related risk of stroke to patients (21.7%) were three dominant causes for failing anticoagulant usage. In conclusion, most AF patients were with a high risk of thrombosis and a low risk of bleeding in China, but a large majority of them failed to take anticoagulants mainly for missing an early screening of AF and lack of awareness on AF for both patients and primary care physicians.

## Introduction

Atrial fibrillation (AF) is the most common arrhythmia in the elderly, with a prevalence of 1–2% in Western populations. The number of AF patients is expected to increase 2.5-fold over the next 50 years in the aging population^1^. Previous studies indicated the prevalence of AF varied greatly among different populations and regions in China^2–6^.

Cardiogenic cerebral embolism has been perceived as the most common and severe complication of AF, which confers high rates of mortality and disability^7^. AF increased the risk of stroke by 4–5-fold^1^ and 25.4% of patients with ischemic stroke had AF recorded during admission^8^. Oral anticoagulants (OAC) played a crucial role in prophylaxis of AF-related stroke. It was reported that adjusted-dose warfarin reduced the risk of stroke by over 60%^9^. Therefore, anticoagulation treatment was recommended to AF patients by both the European Society of Cardiology (ESC) and the American Heart Association (AHA) guidelines^1,10^. However, a study published in 2008 indicated that more than 97% of Chinese AF patients did not take anticoagulants^2^. This finding highlighted a great gap between current guidelines and the clinical management of AF in China. It’s unclear whether this state has been improved over the past decade. The non-vitamin K antagonist oral anticoagulant (NOAC) has been evaluated in large trials for stroke prevention in AF, which indicated NOAC was non-inferior to warfarin in preventing stroke or systemic embolism, with reducing the risk of life-threatening bleeding^1^. The prevalence of antithrombotic treatment in rural populations in Eastern China has not been estimated and there was no study designed to explore the reasons why most AF patients do not take OAC in China.

This study is performed to determine the prevalence of AF in rural populations over 60-years-old in Eastern China and to report the current antithrombotic status of these AF patients. Our study also provides data for analyzing the reasons why anticoagulant usage is so low in Chinese AF patients.

## Methods

### Ethics statement

This study was conducted in accordance with the Helsinki Declaration, and it was approved by the ethics commission of the institutional review board of Shanghai Songjiang Central Hospital, Shanghai, China. All participants were informed of the nature and objectives of the study. Uniform safeguards were provided to protect the confidentiality of personal information. Informed consent was obtained from each enrolled subject.

### Study population and AF screening

**Figure 1:**
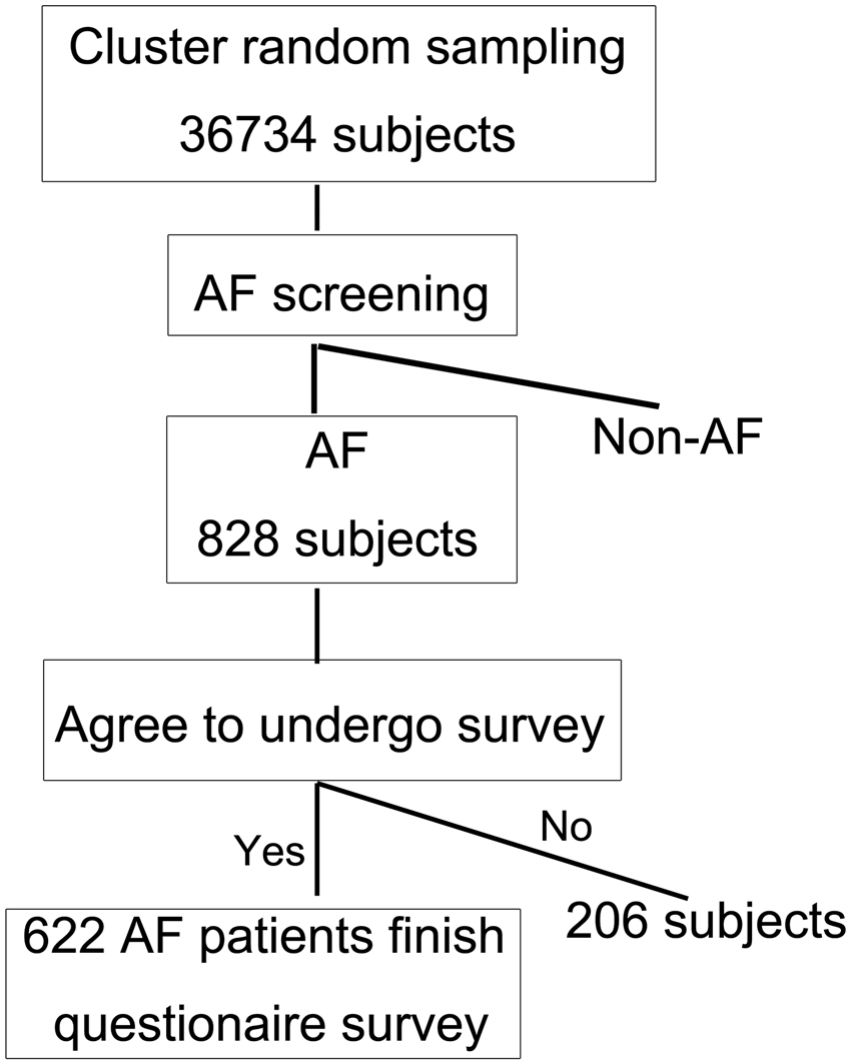
The consort diagram of the study population. AF, atrial fibrillation.

This was a cross-sectional study on the prevalence of AF in rural populations greater than 60-years-old in Eastern China, which involved 36734 subjects from seven rural towns (Xinbang, Maogang, Chedun, Dongjing, Yexie, Shihudang, Xiaokunshan) in Shanghai China. The data for this study were collected from 1st May 2015 to 31st October 2015 in a community health screening project for elderly residents in Shanghai China. All authors listed in this study were the investigators for the Chronic Cardiovascular Disease Management Group in Songjiang Shanghai China. Annual physical examinations were freely supported by the local government to permanent residents who were older than 60-years-old and lived in the aforementioned towns with rural household registration. Cluster random sampling was applied in the screening process. Within clusters, we randomly selected subjects by means of random number table. Protocols for this study are presented in Fig. 1. The inclusion criteria of the study were as follows: (1) aged >60 years old, (2) residents living in the 7 above-mentioned towns with rural household registration, and (3) consenting to take annual physical examinations freely supported by the local government. Those who did not meet all inclusion criteria were excluded. Enrolled sujects were drawn to undergo door-to-door census surveys and invited to participate in a health examination. Twelve-lead electrocardiograms (ECG) were performed on all participants. AF was defined as a cardiac arrhythmia with the following characteristics: (1) The surface ECG showed ‘absolutely’ irregular RR intervals; (2) There were no distinct P waves on the surface ECG; (3) The atrial cycle length (when visible), i.e. the interval between two atrial activations, was usually variable and <200 ms (>300 bpm). AF diagnoses were independently confirmed by two cardiologists.

### Survey of antithrombotic treatment in AF patients

All interviewers got a one-week training session, including clinical aspects of AF and the theoretical and practical aspects of the study design and purpose. And then they were assessed in supervised interviews. The eligible AF patients underwent a questionnaire survey, answering whether they were receiving antithrombotic treatment and addressing what kind of agents they were taking, such as warfarin, aspirin, clopidogrel, or NOAC (dabigatran, rivaroxaban, and apixaban). A risk stratification of stroke and assessment of bleeding risk associated with anticoagulation were performed. CHA_2_DS_2_-VASc [congestive heart failure, hypertension, age ≥75-years-old (doubled), diabetes mellitus, history of stroke (doubled), vascular disease, age 65–74, and sex (female)] score was calculated to estimate the risk of stroke in AF patients^1^. A well-established risk score termed HAS-BLED [hypertension, abnormal renal/liver function, stroke, bleeding history or predisposition, labile international normalized ratio, elderly (>65 years old), drugs/alcohol concomitantly] was calculated to predict the bleeding risk of AF patients who took anticoagulant^1^. HAS-BLED score was assessed for all eligible AF patients. Potential causes as to why AF patients failed to take OAC in China are listed in Table 1. It included five main items and eleven options each participant could select. AF patients who had not taken OAC were asked to select their top reasons.

**Table 1:** Causes on the failure of warfarin usage in atrial fibrillation patients.

Causes	Cases	Percentage (%)
1. Patients did not know they had AF	149	25.5
2. Patients knew that they had AF, but did not seek treatment or see a cardiologist due to lack of symptoms	204	34.9
3. Patients knew that they had AF, for which they went to see a doctor, but were not informed that AF was accompanied with high risks of cerebral embolism and they needed antithrombotic treatment	127	21.7
4. AF patients had been informed that high risks of cerebral embolism were associated with AF and they needed antithrombotic treatment, but warfarin was not suggested by the doctor	83	14.2
4.1 Warfarin was not recommended for the doctor’s worry about the bleeding risk associated with warfarin, but without bleeding risk assessment	53	9.1
4.2 Bleeding risk assessment was performed and indicated a high risk of bleeding associated with warfarin	7	1.2
4.3 Patients with poor compliance, did make an appointment to accept INR monitoring	17	2.9
4.4 Patients previously underwent warfarin treatment, but ceased due to labile INR and the incidence of bleeding events	3	0.5
4.5 Contraindications	3	0.5
5. AF patients had been informed by the doctor that high risks of cerebral embolism were associated with AF and they needed antithrombotic treatment with warfarin, but they rejected to take warfarin	22	3.8
5.1 Concern over bleeding risk associated with warfarin	5	0.9
5.2 Could not comply INR monitoring monthly	9	1.5
5.3 Others	8	1.4
Total	585	

### Statistical analysis

Independent double data entries were conducted via EpiData 3.1 software by two professional data managers. All statistical analyses were performed using SPSS13.0. For continuous variables, expressed as mean ± standard deviation, differences among groups were evaluated by an unpaired t-test or ANOVA. Discrete variables, expressed as counts and percentages, were analyzed by a chi-square or Fisher exact test. Rank sum test was used for ranked data. P < 0.05 was considered statistically significant.

## Results

### The prevalence of AF in the rural population over 60-years-old in Eastern China

**Figure 2:**
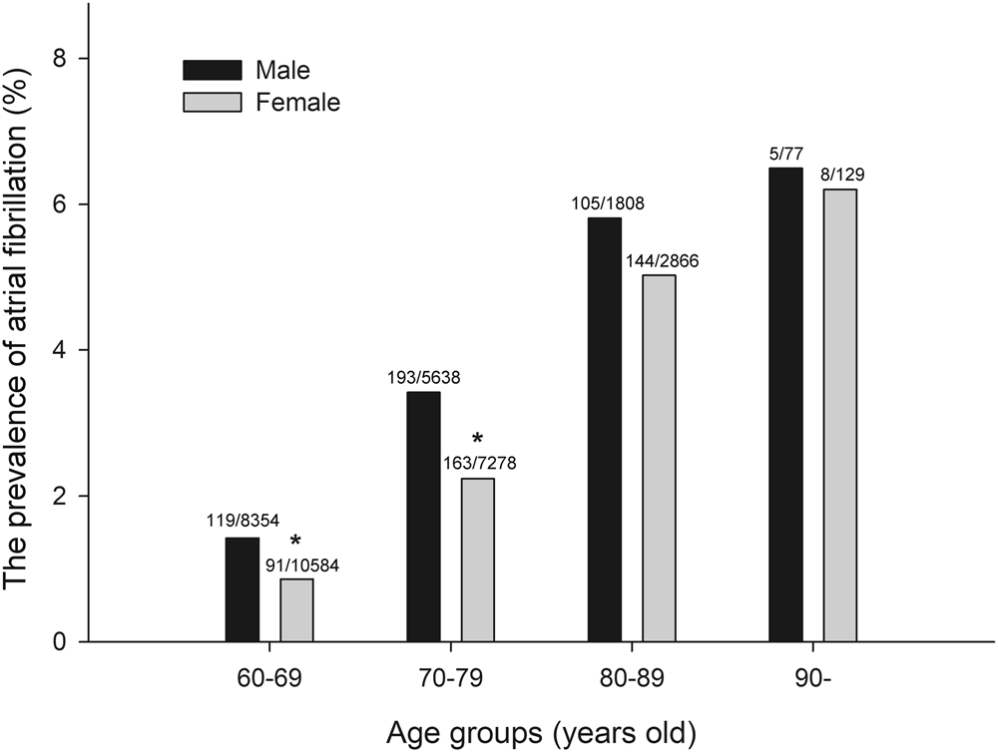
The prevalence of atrial fibrillation in various age groups. Compared with the males in the same age group, *Indicated P < 0.001.

36734 individuals were randomly investigated in seven rural towns in Shanghai China. They included 15877 males and 20857 females. 828 AF patients were selected, including 422 males and 406 females. The overall prevalence rate of AF was 2.3% in rural populations over 60-years-old in Eastern China. The morbidity of AF was higher in males (2.7%) than in females (2.0%, P < 0.001). The prevalence of AF increased gradually with age for both females and males, from 0.9–1.4% in patients 60–69-years-old to 6.2–6.5% in patients ≥90-years-old (Fig. 2). Males had a higher AF prevalence rate than females in both the 60–69 and 70–79 age groups (Fig. 2).

### Current antithrombotic status in rural AF patients over 60-years-old

AF patients were invited to finish the questionnaire survey, which was designed to investigate the current antithrombotic treatment they underwent. Around 25% (206/828) of the screened AF patients did not take part in this epidemiological survey. Seventy-eight subjects refused survey due to their worries about personal privacy being leaked, 76 subjects for having no time to receive questionnaires, 22 subjects for too many questions in the questionnaire, 23 subjects for asserting that it was pointless to participate in the study, and 7 subjects for other unknown reasons. Finally, 622 AF patients (622/828, 75.1%) voluntarily completed the questionnaires. Table 2 shows the clinical characteristics of rural AF patients over 60-years-old in Eastern China.

**Table 2:** Clinical characteristics of patients with atrial fibrillation.

Variable	Total	Male	Female	P*
**Number**	622	314	308	—
**Age, years old**	75 ± 8	74 ± 7	76 ± 8	0.001
**NYHA heart failure class**				0.832
I, n(%)	525(84.4)	264(84.1)	261(84.7)	
II, n(%)	79(12.7)	41(13.1)	38(12.3)	
III, n(%)	16(2.6)	9(2.8)	7(2.3)	
IV, n(%)	2(0.3)	0(0.0)	2(0.6)	
**Body weight index, kg/m** ^**2**^	23.3 ± 3.7	23.2 ± 3.4	23.5 ± 4.0	0.070
**SBP, mmHg**	129 ± 13	130 ± 13	129 ± 13	0.603
**DBP, mmHg**	79 ± 7	79 ± 8	78 ± 7	0.045
**Heart rate, bpm**	80 ± 10	80 ± 11	79 ± 10	0.328
**Education status**				<0.001
Primary school, n(%)	565(90.8)	265(84.4)	300(97.4)***	
Junior school, n(%)	55(8.8)	47(15.0)	8(2.6)	
High school, n(%)	2(0.3)	2(0.6)	0(0.0)	
**Smoking status**				<0.001
Never, n(%)	433(69.6)	141(44.9)	292(94.8)**	
Current, n(%)	78(12.5)	70(22.3)	8(2.6)	
Former, n(%)	111(17.8)	103(32.8)	8(2.6)	
**Drinking status**				<0.001
Never, n(%)	498(80.1)	200(63.7)	298(96.8)****	
Less than one time per week, n(%)	60(9.6)	56(17.8)	4(1.3)	
1 to 7 times per week, n(%)	51(8.2)	46(14.6)	5(1.6)	
More than 7 times per week, n(%)	13(2.1)	12(3.8)	1(0.3)	
**Pharmacological cardioversion, n(%)**	12(1.9)	8(2.5)	4(1.3)	0.258
**AF catheter ablation, n(%)**	3(0.5)	1(0.3)	2(0.6)	0.987
**Pacemaker implantation, n(%)**	17(2.7)	7(2.2)	10(3.3)	0.436
**Coronary heart disease**	233(37.5)	123(39.2)	110(35.7)	0.373
CABG	3(0.5)	2(0.6)	1(0.3)	1.000
CAG	14(2.3)	10(3.2)	4(1.3)	0.113
PCI	4(0.6)	2(0.6)	2(0.6)	1.000
Current angina pectoris	35(5.6)	11(3.5)	24(7.8)	0.020
Previous myocardial infarction	11(1.8)	6(1.9)	5(1.6)	0.786
**Hypertension, n(%)**	371(59.6)	189(60.2)	182(59.1)	0.780
**Diabetes, n(%)**	73(11.7)	31(9.9)	42(13.6)	0.145
**Valve implantation, n(%)**	9(1.4)	3(1.0)	6(1.9)	0.483
**Peripheral artery disease, n(%)**	3(0.5)	2(0.6)	1(0.3)	1.000
**Complex aortic plaque, n(%)**	3(0.5)	3(1.0)	0(0.0)	0.254
**Tumor, n(%)**	20(3.2)	15(4.8)	5(1.6)	0.026
Metastatic, n(%)	3(0.5)	3(1.0)	0(0.0)	0.254
None-metastatic, n(%)	17(2.7)	12(3.8)	5(1.6)	0.093
**Chronic digestive disease, n(%)**	54(8.7)	30(9.6)	24(7.8)	0.435
**Hyperthyroidism, n(%)**	10(1.6)	2(0.6)	8(2.6)	0.104
**Dyslipidemia, n(%)**	87(14.0)	37(11.8)	50(16.2)	0.110
**Hepatic dysfunction, n(%)**	10(1.6)	2(0.6)	8(2.6)	0.104
**Undergoing cardiovascular drugs, n(%)**	419(67.4)	206(65.6)	213(69.2)	0.345
ARB, n(%)	171(27.5)	85(27.1)	86(27.9)	0.812
ACEI, n(%)	25(4.0)	15(4.8)	10(3.2)	0.331
Diuretics, n(%)	99(15.9)	41(13.1)	58(18.8)	0.049
β-receptor blocker, n(%)	130(20.9)	55(17.5)	75(24.4)	0.036
CCB, n(%)	118(19.0)	61(19.4)	57(18.5)	0.770
Digoxin, n(%)	71(11.4)	30(9.6)	41(13.3)	0.141
Other anti-hypertensive agents, n(%)	59(9.5)	28(8.9)	31(10.1)	0.625
Other anti-arrhythmic agents, n(%)	8(1.3)	4(1.3)	4(1.3)	1.000
**Undergoing lipid-lowering treatment**	108(17.4)	49(15.6)	59(19.2)	0.242
Statin	106(17.0)	48(15.3)	58(18.8)	0.240
Other lipid-lowering agents	2(0.3)	1(0.3)	1(0.3)	1.000
**Undergoing other drugs**	88(14.2)	47(15.0)	41(13.3)	0.553

NYHA, New York Heart Association; SBP, systolic blood pressure; DBP, diastolic blood pressure; AF, atrial fibrillation; CABG, coronary artery bypass grafting; CAG, coronary angiography; PCI, percutaneous coronary intervention; ARB, angiotensin receptor blocker; ACEI, angiotensin converting enzyme inhibitor; CCB, calcium channel blocker. *P values for comparing the males with the females.

**Figure 3:**
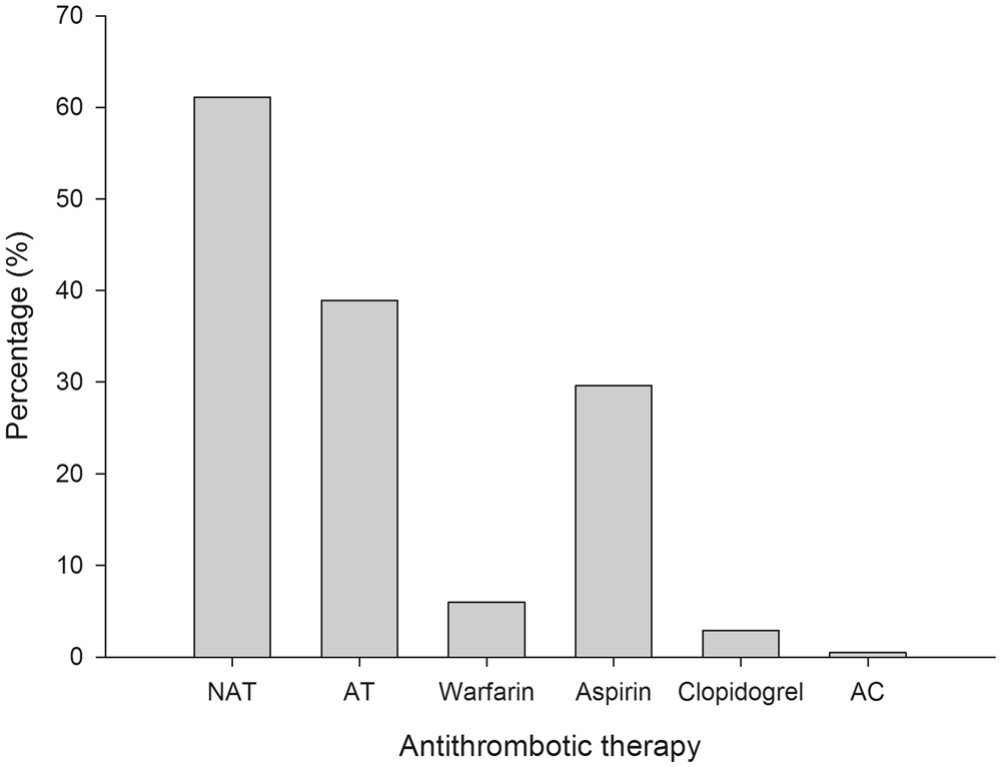
Current antithrombotic status in rural atrial fibrillation patients over 60-years-old. NAT, no antithrombotic; AT, antithrombotic; AC, aspirin combined with clopidogrel.

Of the 622 AF patients, 380 subjects (61.1%) did not receive any antithrombotic treatment. The other 242 patients (38.9%) were presently undergoing antithrombotic therapy, including aspirin (29.6%), warfarin (5.9%), clopidogrel (2.9%), and aspirin combined with clopidogrel (0.5%) (Fig. 3). No patients took NOAC, such as dabigatran, rivaroxaban, or apixaban. Thirty-seven subjects were currently taking warfarin with a mean dosage of 2.1 ± 0.9 mg/day, including 7 patients to whom warfarin was prescribed for valve implantation before the diagnosis of AF. Therefore, warfarin was initially prescribed to only 30 patients (4.8%) for AF

### Risk stratification for stroke and assessment of bleeding risk associated with anticoagulation

**Figure 4:**
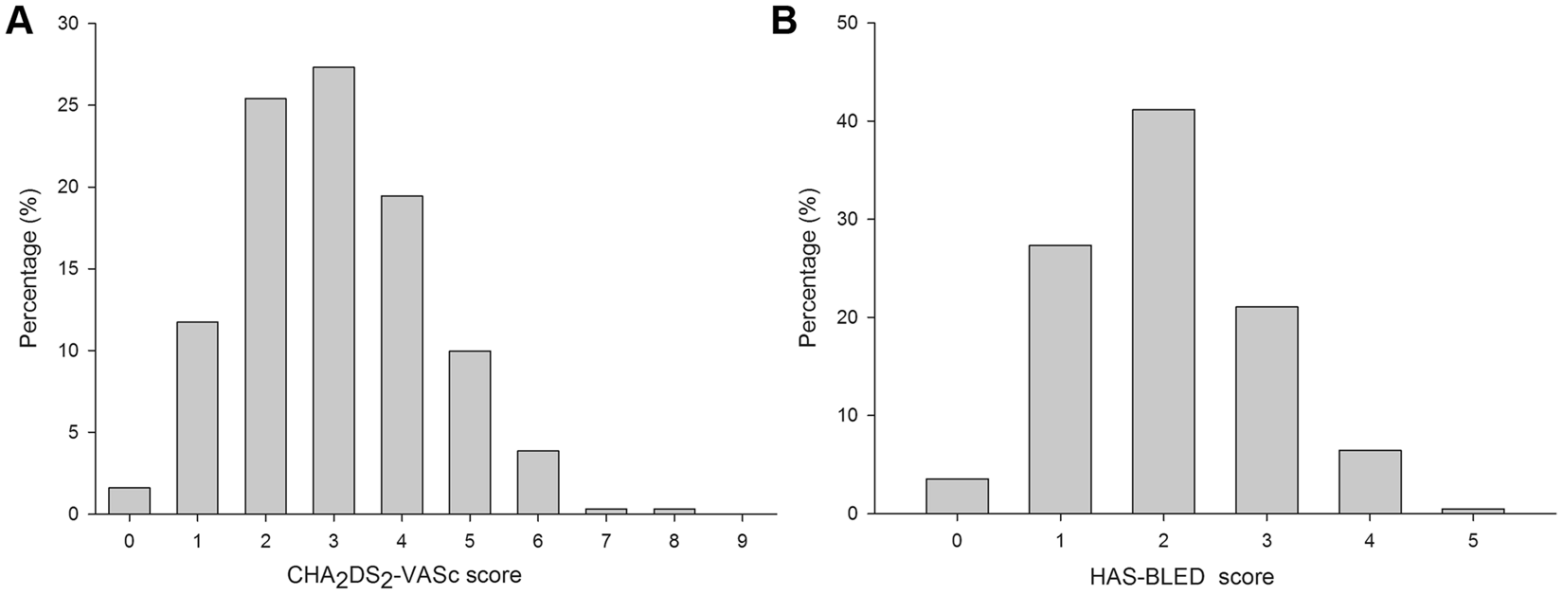
Risk stratification for stroke and assessment of bleeding risk associated with anticoagulation in rural atrial fibrillation (AF) patients. (**A**) CHA_2_DS_2_-VASc score in AF patients; (**B**) HAS-BLED score in AF patients.

622 rural AF patients over 60 years old underwent risk stratification for stroke by CHA_2_DS_2_-VASc scheme. We found that 98.4% of the enrolled subjects had CHA_2_DS_2_-VASc scores ≥1, with CHA_2_DS_2_-VASc scores of one comprising 11.7%, scores of two comprising 25.4%, scores of three comprising 27.3%, scores of four comprising 19.5%, and scores of five comprising 10.0% (Fig. 4A). Our results indicated that 72.0% of the enrolled subjects had HAS-BLED scores <3 (Fig. 4B). Combined assessment of risk stratification for stroke (CHA_2_DS_2_-VASc) and bleeding risk with anticoagulation (HAS-BLED) in AF patients indicated the top three combinations were a HAS-BLED score of 2 with a CHA_2_DS_2_-VASc score of 3 (12.5%), a HAS-BLED score of 2 with a CHA_2_DS_2_-VASc score of 4 (11.9%), and a HAS-BLED score of 1 with a CHA_2_DS_2_-VASc score of 2 (11.3%). Our data showed that 59.2% of patients had both CHA_2_DS_2_-VASc scores ≥2 and HAS-BLED scores <3.

### Analysis of causes for lack of anticoagulation treatment

585 patients had not taken warfarin. Analysis as to why these patients were not taking warfarin is listed in Table 1. The top cause was that patients knew that they had AF but did not seek treatment or see a cardiologist due to a lack of symptoms, accounting for 34.9%. Missing an early diagnosis of AF was the second cause, accounting for 25.5%. Another 21.7% of AF patients failed to undergo warfarin treatment because they were not adequately informed of the AF-related risk of stroke. Only 3.8% of patients refused warfarin treatment, despite having been informed by a doctor that high risks of cerebral embolism were associated with AF and they should undergo antithrombotic treatment.

## Discussion

This study had four highlights: (1) age and sex-related differences in AF prevalence were determined, (2) it highlighted the poor anticoagulation management of AF and the absence of the novel therapies (NOAC) in the field of AF anticoagulation management, (3) it showed that our studied population had a high thromboembolic risk along with low bleeding risk, and (4) it was the first study designed to explore why most AF patients did not receive antithrombotic treatment with warfarin in China, indicating that the top three causes for limited warfarin usage were missed early detection of AF, delays in seeking treatment for asymptomatic AF, and that the patients were often not informed of the AF-related risk of stroke.

AF tended to be less prevalent in Asians than Caucasians^11^. The estimated overall AF prevalence in elderly patients greater than 60-years-old varied among different Asian populations, such as 1.84% (663/36120) in Japanese^12^, 1.59% (80/5044) in Korean^13^, 1.31% (24/1839) in Singaporean^14^, and 2.2% (21/963) in Thai^15^. For Chinese, AF prevalence also varied greatly among different regions and populations, ranging from 0.96% (116/12100) in the general population of Southern China (Guangzhou) to 3.16% (54/1709) in rural populations in Northern China (Liaoning)^6^. Our study firstly reported the prevalence of AF in the rural elderly population in Eastern China, whose sample size was the largest of all studies on AF prevalence in China. We demonstrated that the overall prevalence rate of AF was 2.25% (828/36734) in elderly rural populations in Eastern China. Our data also showed the prevalence of AF steadily increased with age from 0.9–1.4% in the 60–69 age group to 6.2–6.5% in the ≥90 age group, which was similar to those obtained for Western populations^16^. Previously, some studies indicated there were no significant differences in AF prevalence between men and women in China^5,6^, while others suggested that the prevalence of AF was higher in males than in females^2^. The difference in AF prevalence between men and women was observed in our study. We confirmed that AF was more prevalent in elderly men than in elderly women. In both the 60–69 and 70–79 age groups, males had a higher AF morbidity than females. Another cross-sectional study indicated that the prevalence of AF was 3.3% in 859 elderly men and 3.1% in 850 elderly women (≥65-years-old), who were rural residents of Northern China (Liaoning Province)^6^. It evoked our interest in whether differences in AF prevalence existed between Eastern and Northern populations in China. Our data show that the prevalence of AF was 2.9% (395/13670) for males and 2.04% (386/18916) for females in the elderly population (≥65-years-old) of rural residents. Comparing this study with ours, we found that AF prevalence was higher in Northern females than in Eastern females, with no difference between Eastern and Northern rural males. Reasons for the difference in AF prevalence between different Chinese populations are unclear. We hypothesize that it is mainly due to the difference in hypertension and heart disease prevalence. For instance, the morbidity of hypertension in Northern China is significantly higher than that in Southern China^17^ and hypertension is the most important risk factor for AF.

AF-induced atrial contractile dysfunction precipitates thrombus formation in the atria, which can subsequently break off and cause stroke or systemic embolism. Therefore, antithrombotic treatment is critical for most AF patients according to CHA_2_DS_2_-VASc scoring. Anticoagulation with warfarin significantly reduced the risk of AF-induced stroke by 64%, while aspirin reduced it by only 22%^9^. Several studies reported that NOAC, such as dabigatran, rivaroxaban, and apixaban, were powerful in reducing the risk of stroke and systemic embolism, without requiring intensive monitoring and complex dose adjustment^18–20^. Dabigatran is approved for anticoagulation treatment in AF patients in China, but its current cost (20 RMB per 110 mg, 1200 RMB per month) is pretty high and far beyond economic affordability for most AF patients in China, especially for rural individuals, whose average gross monthly earnings are about 1500 RMB. Cost is a major barrier to NOAC use for Chinese rural AF patients. Therefore, we anticipate that warfarin is still the first-choice anticoagulant for most rural patients with AF in the next decades in China. Our data indicate that the current management of AF-related stroke is still poor in rural Chinese populations, with low awareness, under-use, or no use of anticoagulants. Only 5.9% of rural AF patients over 60-years-old underwent anticoagulation therapy with warfarin and none took NOAC. Compared with warfarin, anti-platelet agents (mostly aspirin, 29.58%) were more frequently prescribed to AF patients in China. The global anticoagulant registry in the FIELD (GARFIELD) indicated 60.3% of non-valvular AF patients received anticoagulants for stroke prevention, including 55.8% of whom were given a vitamin K antagonist (VKA) and 4.5% received a NOAC^21^. Subgroup analysis of the GARFIELD registry demonstrated only 28.7% of Chinese non-valvular AF patients received either warfarin (22.2%) or NOAC (6.5%)^22^. However, our data showed only 5.9% of rural AF patients took OAC. Chinese AF patients studied in the GARFIELD registry were prone to citizens, for they were enrolled at the hospitals that located in big cities in China. But we studied AF patients screened from the general rural population, who were with poor economic and medical conditions and lack of knowledge about prevention and treatment of AF. Great difference is also observed in anticoagulation therapy for AF in rural areas between China and Western countries. Compared with our study, anticoagulant dispensation for AF patients residing in the rural areas was higher in Western countries, such as Ireland (64.7%)^23^ and Canada (72.2%)^24^. None of the AF patients in our study received NOAC, which is in contrast with the Irish^23^ and Greek study^25^. The high cost impairs patients to adhere to NOAC therapy, which is not affordable for the great majority of AF patients in China. And it subsequently impairs the willingness of the clinicians to prescribe NOAC and only a few of hospitals are willing to provide NOAC. In the Greek study, acenocoumarol was used as the only VKA instead of warfarin^25^. However, acenocoumarol is not a well-known VKA in China. For most Chinese primary hospitals, the only available OAC is warfarin rather than acenocoumaroin. A Canadian population based study showed there was no difference in warfarin dispensation between rural and urban non-valvular AF patients (rural 72.2% vs. urban 71.8% at 365 days, p = 0.98) for they were in a universal access publically-funded healthcare system^24^. However, rural residents have lower health awareness, poor social support and education and less compliance with close INR monitoring called for warfarin titration due to the lack of easily accessible medical care in many rural areas in China. Therefore, we hypothesize that great gap of anticoagulant therapy for AF exists between urban and rural populations in China.

It was unknown why most AF patients did not undergo anticoagulation in China. Our study indicated that missing early detection of AF and delays in seeking treatment for asymptomatic AF were two dominant causes for limited warfarin usage. Another salient reason was that doctors did not adequately inform patients of the AF-related risk of stroke. Only 3.76% of AF patients rejected to take warfarin after being informed of the high risk of cerebral embolism associated with AF by the doctor. It indicates that the low percentage of patients under anticoagulation therapy is a consequence of the limited access to the healthcare services and problems to the health care system instead of the patients’ preferences. Accordingly, to improve the application of warfarin in such patients, greater effort should be taken for the early identification of AF through awareness-raising campaigns coupled with targeted screening programs and AF-related education for both physicians and patients. We are developing a patient-centered, family-supported, community doctor-led, guideline-based chronic care approach for Chinese AF patients (ChiCTR-ICR-15007036) to provide appropriate management of INR monitoring and dose-adjustment. The effects of this program on management of AF-related stroke remain to be determined.

### Limitations

First, our data indicated a significant percentage of AF patients had HAS-BLED score <3. This is strange as the elderly people usually have the high thromboembolic risk along with the high bleeding risk. The limited access of these patients to the health care services has a result not only to receive subtherapeutic antithrombotic treatment for AF but also to have comorbidities such as hypertension/renal disease/and labile INR under-diagnosed. Second, this is a study which took part in a rural and low-income region of East China. The low proportion of AF patients treated with anticoagulants can be changed if NOAC becomes affordable for the vast majority of patients in our region. AF patients may present a better compliance with NOAC for no INR monitoring, no diet instructions and less hemorrhagic strokes. Third, we didn’t categorize the patients according to their AF profiles (paroxysmal AF, persistent AF or permanent AF) and such data were not available. Although the type of AF does not play any role in the anticoagulation management of AF till now, it is important as AF burden may have significant clinical implications in the future. Finally, this study likely underestimated AF prevalence, as AF was determined by single ECG recordings. It’s difficult to perform continuous 24-hour ambulatory ECGs for AF screening in a larger survey. Therefore, some cases of paroxysmal AF might be missed. The prevalence of AF in this age group is low and it’s in contrast with the latest surveys/studies of AF which refer that the prevalence of AF is greater than 10%. However, this can be attributed to the limited access of the rural Chinese population to health care services.

## Conclusions

We confirm that the prevalence of AF increases dramatically with age and males tend to have a higher AF prevalence than females in the elderly rural population in Eastern China. The use of anticoagulants in such patients is limited. Missing an early diagnosis of AF, delays in seeking treatment for asymptomatic AF, and limited understanding of the AF-related risk of stroke are the three dominant causes for limited warfarin usage. Great efforts should be taken to assure the early identification of AF and increase the awareness of AF for both patients and primary care physicians.
